# Stages of Change – Continuous Measure (URICA-E2): psychometrics of a Norwegian version

**DOI:** 10.1111/j.1365-2648.2008.04842.x

**Published:** 2009-01

**Authors:** Anners Lerdal, Britt Moe, Elin Digre, Thomas Harding, Frode Kristensen, Ellen K Grov, Linda N Bakken, Marthe L Eklund, Ireen Ruud, Joseph S Rossi

**Affiliations:** Anners Lerdal PhD RN Associate Professor Department of Health Sciences, Buskerud University College, Drammen Researcher Research Centre, Aker University HospitalOslo, Norway; Britt Moe RN Assistant Professor Department of Health Sciences, Buskerud University CollegeDrammen, Norway; Elin Digre MSc RN Associate Professor Department of Health Sciences, Buskerud University CollegeDrammen, Norway; Thomas Harding PhD RN Associate Professor Department of Health Sciences, Buskerud University CollegeDrammen, Norway; Frode Kristensen MSc RN Associate Professor Department of Health Sciences, Buskerud University CollegeDrammen, Norway; Ellen K. Grov PhD RN Associate Professor Department of Health Sciences, Buskerud University CollegeDrammen, Norway; Linda N. Bakken MSc RN Research Fellow Department of Health Sciences, Buskerud University CollegeDrammen, Norway; Marthe L. Eklund MSc RN Assistant Professor Department of Health Sciences, Buskerud University CollegeDrammen, Norway; Ireen Ruud MSc RN Assistant Professor Department of Health Sciences, Buskerud University CollegeDrammen, Norway; Joseph S. Rossi PhD Professor Cancer Prevention Research Centre and Department of Psychology, University of Rhode IslandKingston, USA

**Keywords:** exercise, health promotion, instrument validation, Norwegian, psychometrics, Stages of Change – Continuous Measure (URICA-E2), Transtheoretical Model

## Abstract

**Title:**

**Stages of Change – Continuous Measure (URICA-E2): psychometrics of a Norwegian version.**

**Aim:**

This paper is a report of research to translate the English version of the Stages of Change continuous measure questionnaire (URICA-E2) into Norwegian and to test the validity of the questionnaire and its usefulness in predicting behavioural change.

**Background:**

While the psychometric properties of the Stages of Change categorical measure have been tested extensively, evaluation of the psychometric properties of the continuous questionnaire has not been described elsewhere in the literature.

**Method:**

Cross-sectional data were collected with a convenience sample of 198 undergraduate nursing students in 2005 and 2006. The English version of URICA-E2 was translated into Norwegian according to standardized procedures.

**Findings:**

Principal components analysis clearly confirmed five of the dimensions of readiness to change (Precontemplation Non-Believers, Precontemplation Believers, Contemplation, Preparation and Maintenance), while the sixth dimension, Action, showed the lowest Eigenvalue (0·93). Findings from the cluster analysis indicate distinct profiles among the respondents in terms of readiness to change their exercise behaviour.

**Conclusion:**

The URICA-E2 was for the most part replicated from Reed’s original work. The result of the cluster analysis of the items associated with the factor ‘Action’ suggests that these do not adequately measure the factor.

**What is already known about this topic**

- A critical issue in current health promotion is the apparent decrease in people’s daily physical activities and exercise.
- Sedentary lifestyle has been linked to various health problems such as obesity and musculoskeletal weakness, and influences the management of many chronic illnesses such as cardiovascular diseases, diabetes and depression.
- Changing people’s exercise behaviours and helping them to maintain health-promoting exercising behaviour patterns are critical for health.

**What this paper adds**

- The psychometric properties of the Norwegian version of the URICA-E2 were for the most part replicated from the original work and thus are consistent with the theoretical underpinnings of the instrument.
- Further validation of the Action items of the Norwegian version of the URICA-E2 and its concurrent validity is required if this instrument is to be used in intervention studies.
- The URICA-E2 vs. the Stages of Change categorical measure might have the potential to identify individuals’ change profiles in the different stages.

**Implications for practice and/or policy**

- The study provides an assessment tool for exercise lifestyle change.

## Introduction

Physical activity is one of the main behaviours or parts of the lifestyle in promoting health. Norway, like many other countries, is concerned about the decreased level of physical activity in the population in recent decades (Ministry of Social Affairs 2002–2003). It is well-documented that a moderate to high level of physical activity prevents physical illness in addition to increasing individual fitness (Haskell *et al.* 2007). Studies have also shown positive effects of moderate physical activity on mental health (Larun *et al.* 2006).

The Transtheoretical Model (TTM) of behavioural change has been used in numerous studies throughout the world (e.g. Buckworth & Wallace 2002, Cardinal *et al.* 2004, Prapavessis *et al.* 2004) and is cited as one of the most important theoretical models for describing processes of behavioural change in the field of health promotion (Samuelson 1997). The model was first described in early 1980s, when it was applied to smokers to compare the processes of change in cessation and maintenance (DiClemente & Prochaska 1982). Subsequently, application of the model has been widened to describe behavioural changes among individuals with health problems such as alcohol and substance abuse, anxiety and panic disorders, and diet (Prochaska & Velicer 1997, Helitzer *et al.* 2007, Hall & Rossi 2008). In addition to its use in studies of behavioural change related to risk behaviours, it has also been applied in health promotion studies, e.g. in intervention studies aimed at increasing levels of physical activity and motivating individuals to start exercising regularly (Lowther *et al.* 2007).

An important concept in the TTM is the Stages of Change (SoC), which describes the phases through which an individual passes when changing health behaviour. Readiness to change consists of a continuum of five stages: from Precontemplation, through Contemplation, Preparation, and Action to Maintenance (Nigg 2005). The original model included six stages, with the last being Termination (Prochaska 2003). This last stage was not applied in the measurement related to this study because changes in exercise behaviour such as adherence to exercise is problematic, with up to 50% of those who start exercise programmes quitting after 1 year (Dishman 1988). With exercise, it appears difficult to reach the stage of termination (Reed 1993), and up to 72% of people lapse to a previous stage (Berry *et al.* 2005).

A categorical measure of SoC has been developed and used in several studies of different risk behaviours and motivation to exercise regularly (Reed *et al.* 1997, Buckworth & Wallace 2002, Cardinal *et al.* 2004). The high rates of relapse shown in studies aimed at increasing physical activity may indicate that motivation for performing and actual performing of physical activity are more unstable and fluctuating phenomena than, for example, processes related to smoking cessation. It is assumed that an individual in one stage may have feelings and thoughts also from other stages. To capture these characteristics, a continuous measure of Change of Stages of Exercise – University of Rhode Island Change Assessment, the third generation (URICA-E2), has been developed. In this instrument, respondents rate the degree to which they are in the different stages of change. There is also a categorical measure specifically developed and applied in various research studies for the stages of change for exercise – Exercise-Stages of Change: Short Form – which has been examined for its validity and reliability (Nigg *et al.* 2005, Hellsten *et al.* 2008).

Interest in the URICA-E2 is in using a continuous scale that could capture the structure of stages of change for exercise behaviours, which seem to be somewhat more complex than those in other behaviours such as smoking and diet (Reed 1995). An evaluation of psychometric properties in relation to the theoretical assumptions of the URICA-E2 is needed.

The English version of the URICA-E2 is available on internet, published by the University of Rhode Island Cancer Prevention Research Centre (Cancer Prevention Research Center 2007). Although Reed (1995), in an unpublished doctoral thesis, tested the psychometric properties of this instrument, to our knowledge, no study has so far been published.

## The study

### Aim

The aim of this study was to translate the American English version of the Stages of Change – Continuous Measure into Norwegian and to test the psychometric properties of this version.

### Methodology

The data for this study was obtained as a part of a study examining relationships among the stages of change constructs, lifestyle behaviours including smoking habits, physical activity, sleep, and fatigue in a sample of college students. Cross-sectional data were collected by means of a questionnaire.

### Sample

The sample was a convenience volunteer sample of 198 first-year nursing students enrolled at a Norwegian university college.

### Instrument

The questionnaire consisted of four parts. The first part assessed participants’ age and sociodemographic background. The second part focused on lifestyle behaviour such as smoking habits, general level of physical activity and insomnia. The URICA-E2 was included as the third part, and the last part assessed level of fatigue. Only the data from the first and the third part are reported in this paper.

#### Stages of change

The 24-item continuous measure (URICA-E2) was used to measure the stages of change related to exercise behaviour (Cancer Prevention Research Center 2007). The original URICA measure consisted of a checklist of five stages (Reed 1993). Precontemplation (PC) has been defined as the stage where the individual has no plan to change a certain behaviour for at least 6 months. In the contemplation phase (C), individuals are planning to change their behaviour within the next 6 months. Preparation (P) is the phase where the individuals are planning to change within the next 30 days and have tried out the specific behaviour a couple of times in the last year. When individuals have been practising the specific behaviour for less than 6 months, they are classified as being in the Action phase (A), and in the Maintenance phase (M) when they have practised the behaviour for longer than 6 months. Based on this original URICA measure, the URICA-E extends the categories into six stages, classifying individuals in the PC phase into two sub-groups; Non-Believers (PC-NB) and Believers (PC-B) in the specific healthy behaviour (Reed 1993). Members of these two sub-groups, however, are perceived as not having plans for exercise-related behaviour change within the next 6 months.

Development of URICA-E2 from this categorical measure (URICA-E) was based on the assumption that the behaviour of physical exercise is typically a dynamic process moving back and forth between the different stages. This results in a mixture of attitudes and mind-sets about exercise in individuals at any given time. It is believed that a continuous measure captures the phenomenon in a better way than the five-item algorithm measurement that is developed in the TTM for other behaviour changes (Reed 1993). Responses to each of the URICA-E2’s 24 items are given on a scale of 1–5, with 1 for ‘strongly disagree’, 2 for ‘disagree’, 3 for ‘undecided’, 4 for ‘agree’, and 5 for ‘strongly agree’.

#### Translation of the URICA-E2

Translation of the English version of URICA-E2 was performed according to the procedures describe by Acquadro *et al.* (1996). First, the US-English version was translated into Norwegian by two bilingual translators, focusing on transposition of the items’ meanings. A faculty research group working on the project describing exercise among students enrolled in a university college programme discussed the differences and agreed on a preliminary version; this was then pilot tested with 14 individuals. This was followed by interviews asking each participant in the pilot test about their understanding of each of the 24 items, the wording and if they had any suggestions for revision. They were also asked to comment on the response alternatives. A summary of the interviews was discussed by the faculty research group, who agreed on a final version; this was then back-translated by two bilingual translators into English. No cultural factors other than language were taken into account in the adaptation of the questionnaire for the Norwegian population. The English versions were reviewed by an English-speaking professor, who found the version to be in accordance with the original English version.

### Data collection

The questionnaire was administered at a scheduled lecture to all the first year Bachelor of Nursing students (*n*= 226) at a university college in Norway in 2005 and 2006; 87·6% (*n*= 198) submitted the completed questionnaire anonymously the following week in a box at the Department’s reception desk.

### Ethical considerations

The appropriate ethics committee approved the study and permission was granted by the Norwegian Data Inspectorate. Informed consent was implied by respondents handing in the completed questionnaire after receiving full information about the aim of the study and the voluntary nature of participation. No data which could identify individual students were collected.

### Statistical analysis

Data were analysed using spss for Windows Version 14.0 software (SPSS Inc., Chicago, IL, USA), with the level of statistical significance set at *P*< 0·05. Descriptive statistics were used to analyse the sample characteristics. The reliability of each sub-scale was assessed by Cronbach’s alpha. A principal components analysis (PCA) with varimax rotation was used to examine the structure of the instrument. Several indicators were used to assess the appropriateness of the correlation matrix for analysis, including the Kaiser–Meyer–Olkin (KMO) index of sampling adequacy, Bartlett’s Test of Sphericity, and the determinant of the correlation matrix. The number of factors extracted was based on the scree test and on Horn’s parallel analysis, as well as on the theoretical assumptions of the scale (Lautenschlager 1989, Redding *et al.* 2006). All study participants were then categorized into clusters using a K-means Cluster analysis. In this analysis, standardized T scores were used (Mean = 50, sd = 10) for the mean scores of each stage’s items.

## Results

### Participants

Most participants were women 86% (*n*= 170); their ages ranged from 18 to 50 years (Median = 22·0, sd = 8·6), with a mean of 28·8 years.

### Psychometric properties

Preliminary analyses were conducted to assess the appropriateness of the correlation matrix for dimensional analysis. The KMO index of sampling adequacy was 0·863, Bartlett’s Test of Sphericity was statistically significant, χ^2^ = 276, d.f. = 3063, *P*< 0·001, and the determinant of the correlation matrix was non-zero. All indices indicated that the matrix was adequate (Tabachnick & Fidell 2007).

A PCA was conducted on the inter-item correlation matrix of the 24 URICA-E2 items. Six components were extracted and subjected to varimax rotation based on the theoretical structure of the measure. In addition, both the scree test and Horn’s parallel analysis were conducted to verify the number of components, and both procedures indicated five components. Item loadings are shown in Table 1, along with the item loadings obtained by Reed (1995) in the original analysis of the measure.

**Table 1:** Factor structure of the URICA-E2 (*n*= 198)

Items and factors	Factor 1	Factor 2	Factor 3	Factor 4	Factor 5	Factor 6	PCA†
Factor 1. Precontemplation Non-Believers in exercise							
1. As far as I am concerned, I don’t need to exercise regularly	0·63*	0·06	−0·14	−0·02	0·00	−0·15	0·80
3. I don’t exercise now and I don’t care	0·55*	0·12	0·09	−0·16	−0·13	−0·22	0·75
6. I am satisfied with being a sedentary person	0·78*	0·18	−0·07	−0·02	−0·02	−0·15	0·82
9. I could exercise regularly, but don’t plan to	0·41*	0·35	−0·06	0·02	0·19	−0·08	0·78
Factor 2. Precontemplation Believers in exercise							
11. I don’t have time or energy to exercise regularly right now	0·21	0·75*	0·02	−0·03	−0·07	−0·32	0·83
19. I know that it is worthwhile, but I don’t have time	0·11	0·73*	0·10	−0·01	−0·04	−0·21	0·86
21. Exercise is good, but I can’t fit it into my schedule now	0·09	0·87*	0·12	−0·05	−0·02	−0·30	0·90
24. I’m aware of the importance of regular exercise but can’t do it now	0·16	0·84*	0·05	−0·06	−0·01	−0·32	0·87
Factor 3. Contemplation							
7. Might start exercising regularly	−0·06	0·16	0·65*	0·16	−0·06	−0·12	0·82
13. Thinking whether able to start	0·12	0·15	0·27*	0·13	0·19	−0·25	0·83
16. Thinking about whether to start	0·00	0·11	0·78*	0·16	−0·06	−0·31	0·91
22. Think getting starting within 6 months	−0·23	0·08	0·56*	0·40	−0·10	−0·21	0·91
Factor 4. Preparation							
14. Have set up a time to start in a few weeks	0·02	−0·24	0·23	0·43*	−0·08	0·28	0·70
17. Have organised with a friend to start	−0·02	−0·02	0·19	0·81*	0·04	0·03	0·91
20. Have been calling friends to start	−0·04	−0·01	0·11	0·83*	0·10	0·08	0·90
23. I really think I should work on getting started	−0·09	−0·01	0·17	0·74*	−0·01	−0·13	0·71
Factor 5. Action							
4. I am finally exercising regularly	−0·23	−0·37	−0·15	−0·02	0·25	0·76*	0·75
8. Have started exercising regularly	−0·13	−0·09	0·42*	0·21	0·21	0·31	0·87
10. Recently, have started exercise regularly	−0·03	−0·13	0·58*	0·17	0·40	0·33	0·83
12. Have started and plan to continue	−0·24	−0·44	−0·03	0·09	0·26	0·71*	0·73
Factor 6. Maintenance							
2. Have been exercising for a long time and plan to continue	−0·24	−0·24	−0·09	0·03	0·06	0·82*	0·92
5. Have been successfully exercising and plan to continue	−0·25	−0·39	−0·03	0·03	0·42	0·66*	0·90
15. Have managed to keep exercising the last 6 months	−0·12	−0·26	−0·06	0·01	−0·11	0·82*	0·94
18. Have completed 6 months of regular exercise	−0·12	−0·26	−0·14	−0·00	−0·13	0·89*	0·93
Eigenvalues	1·38	3·94	1·61	1·73	0·93	7·99	–
Explained variance (%)	5·7	16·4	6·7	7·2	3·9	33·3	–
Explained variance (total)	73·2%	–					

PCA, principal components analysis.

† Factor loadings for each item on its designated target factor from a principal components analysis from a study by Reed (1995).

* Items with the highest loading.

**Figure 1:**
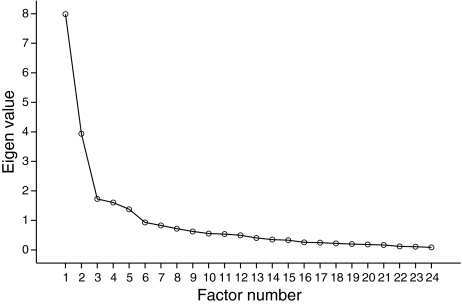
A scree plot illustrating the factor loadings of the 24-item in the URICA-E2.

The six factors accounted for 73% of the variance. This analysis discriminated the 24 items into six conceptual factors that were embedded in the construction of the measure. The first two factors represented the highest proportions of the variance (49·7%), which is illustrated by the steep line related to the first two factors in Figure 1.

The PCA results were used to formulate six components within the URICA-E2, each component consisting of four items on the questionnaire. The four items identified for each of the six factors had the highest loadings for the specific factor, except Factor 5 (Action), which had rather low factor loadings for four items (but higher than any other items on this factor). The items for Factor 5 had higher factor loadings on other factors: items 4 (I am finally exercising regularly) and 12 (I have started exercising and plan to continue) loading highly on Factor 6 (Maintenance), and items 8 (I have started exercising regularly) and 10 (Recently, I have started exercise regularly) on Factor 3 (Contemplation). However, because the conceptual ideas embedded in these four items are more in line with Action than either Contemplation or Maintenance (Factors 3 and 6), we retained these items on the Action factor in subsequent analyses.

Mean scores and alpha coefficients for each of the subscales are shown in Table 2. Contemplation had the highest individually summarized mean raw score, while PC-NB had the lowest mean. This may mean that the respondents were able to express pro-exercise attitudes in a more positive way, whereas they expressed anti-exercise sentiments in a rather neutral way. The analysis showed that the factors that are theoretically close to each other had higher inter-correlation than between those that were more distant (Table 2).

**Table 2:** Means of summarized raw scores, standard deviation, alpha coefficient and correlation for the six scales

Scale	Mean	sd	Alpha	PC-NB	PC-B	C	P	A	M
Precontemplation Non-Believers	6·41	2·70	0·72	1·00					
Precontemplation Believers	9·70	4·87	0·72	0·41	1·00				
Contemplation	13·85	3·90	0·72	0·00	0·27	1·00			
Preparation	10·92	4·55	0·88	−0·16	−0·14	0·48	1·00		
Action	11·29	4·71	0·77	−0·43	−0·46	−0·04	0·30	1·00	
Maintenance	12·48	5·35	0·92	−0·42	−0·61	−0·37	0·11	0·77	1·00

PC-NB, Precontemplation Non-Believers; PC-B, Precontemplation Believers; C, Contemplation; P, Preparation; A, Action; M, Maintenance.

**Figure 2:**
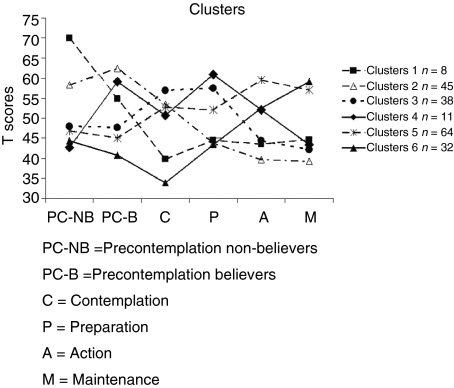
Mean scores for the dimensions in the URICA-E2 for the study participants categorized in six clusters. PC-NB, Precontemplation Non-Believers; PC-B, Precontemplation Believers; C, Contemplation; P, Preparation; A, Action; M, Maintenance.

**Figure 3:**
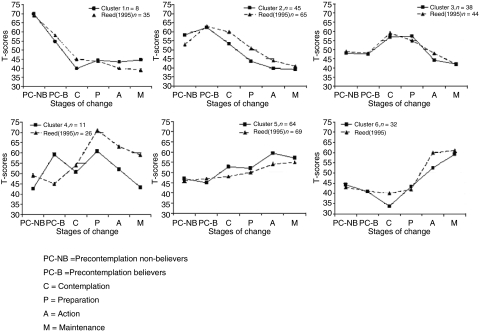
Mean scores for the dimensions in the URICA-E2 from the study participants categorized in six clusters compared with study findings by Reed (1995). PC-NB, Precontemplation Non-Believers; PC-B, Precontemplation Believers; C, Contemplation; P, Preparation; A, Action; M, Maintenance.

Through a K-means cluster analysis, respondents were grouped into six categories according to the factors extracted from this analysis, resulting in eight (4%) in PC-NB, 45 (22·7%) in PC-B, 38 (19·2%) in C, 11 (5·6%) in P, 64 (32·3%) in A, and 32 (16·2%) in M. Mean scores for all six subscales for the six groups are shown in Figure 2. Each cluster was labelled by the stage which showed the highest mean scores. The PC-NB group had the highest mean score on the respective factor, with low mean scores on all the other factors. The remaining five groups had the highest mean scores on the appropriate factors of their categorization, but also had similarly high mean scores on one or two other related factors. The PC-B group had a similarly high mean score on the PC-NB and C clusters, while the C group had a similarly high mean score on the P cluster but not on other clusters. The P group had a similarly high mean score on the PC-B cluster. The Action (A) and Maintenance clusters (M) were similar to each other as both had high mean scores on both factors. These six group mean scores on the different clusters were very similar to Reed’s (1995) study (Figure 3).

## Discussion

This is, to our knowledge, the first published study reporting the psychometric properties of the SoC continuous measure for exercise. The six dimensions identified in the development of the original instrument were in general confirmed by the principal components analysis, even though the loadings are generally lower than those reported in the study by Reed (1995). One underlying idea in the SoC model is that an individual’s readiness to change can be described on a continuum of feelings and thoughts ranging from no interest at all (Precontemplation) through considering (Contemplation, Preparation) to exercising (Action and Maintenance). The correlation analysis between the different stages confirmed this continuum (Table 2), with scores for stages conceptually close to each other showing a higher correlation than between more distant stages.

Our analysis identified five factors that were conceptually in alignment with the theory of the SoC. However, the items originally identified as belonging to the Action stage were somewhat problematic in being categorized as such. Items 4 and 12 had the highest loadings on Factor 6 (Maintenance), and were not high on Factor 5 (Action). The ideas embedded in these items could be interpreted by respondents as ‘keeping up with exercise’, which is the idea underpinning Factor 6. Although we decided to include these items on the Action scale for our other analyses because they are clearly for Action conceptually, the results may be the effects of Norwegian language use. This relates especially to item 4, which in the English version is ‘I am finally exercising regularly’. The word ‘finally’ was left out of the Norwegian version, as the Norwegian word for ‘regularly’ (regelmessig) implies maintenance behaviour. In addition, the high factor loadings of items 8 and 10 on Factor 3, which represents the Contemplation factor, do not seem logical. This might be due to the translation of the questionnaire into Norwegian or by a random effect. In this sense, the factor for Action was most problematic from a statistical position, although the four items which are conceptually most appropriate had the highest loadings on the factor (but these were not statistically significant). This may mean that it is not easy for respondents to strongly agree to exercising currently as it is easy to be in and out of exercising for many reasons. In contrast, it seems that it is not too difficult to agree with the idea that they are in Maintenance (that is, keeping up with exercising). These findings partly support the need to apply a continuous scale such as the URICA-E2 rather than a five-category scale for the stages of change (URI Exercise–Stage of Change: Short Form).

There are several possible additional reasons for the relative weakness of the Action scale. One is the ‘transient’ nature of the Action stage. By its nature (and unlike the other stages of change), individuals can only be in the Action stage for a limited period of time. In many populations (and for many behaviours), the Action stage often contains the fewest individuals. This might result in difficulty in the scale emerging clearly in the analysis. A larger sample would help to answer this issue. In addition, it is possible that individuals in our study simply did not discriminate between the Action and Maintenance stages. If this were the case, then a combined Action/Maintenance scale might be warranted. Only additional research can resolve these issues. However, since most of the previous research on the TTM across a wide range of behaviours clearly supports the distinction between the Action and Maintenance stages (e.g. Hall & Rossi 2008), we considered it best to retain these as two separate scales.

The operational definitions of each stage indicate that one can only be classified into one group at a time. For example, if one is planning to start exercising within 6 months (in the C phase), this is a very different statement from those in the PC phase, who have no plan to change the specific behaviour. When using the URICA-E2 continuous measure, most participants have a scoring profile with highest scores on a specific stage, juxtaposed with some similarly high scores on other subscales. The different scoring profiles in our study seem to support the ‘fluctuating’ aspects of exercise behaviour. As the population matures, the challenge of accurately measuring the moving back and forth, e.g. from Action to Maintenance or from Preparation to Action, might be important for the design and implementation of successful interventions. In this context, the URICA-E2 might serve as an intermediate outcome measure in exercise intervention studies and as an indicator of progress through the stages of change. For example, as a result of an intervention, scores on the Precontemplation and Contemplation scales might be expected to decrease, and scores on the Action and Maintenance scales to increase. Such changes might be seen as precursors to actual change in exercise behaviour.

Since the cluster analysis in our study is very similar to the findings of six clusters by Reed (1995), there might be generic profiles of scores according to the stages that each respondent considers most representative. While the clusters at each end of the stages of change continuum (PC and M) showed much lower mean scores on the other stages, stage C did not. The flat curve in the C cluster can be interpreted as ambivalence, which matches the theoretical definition of the C stage and may be the profile for this stage.

Each stage category of the 24-item URICA-E2 consists of four items which are relatively similar. This may be a limitation of the measure, because respondents may think that they are answering the same question several times. However, the conceptual intention of URICA-E2 as a continuous scale is to fine-tune the stages in order to capture the degree of adherence to the stages and formulate stage-related profiles for in-depth studies of the stages of change in relation to exercise behaviours.

As suggested earlier, exercise behaviour is a more complex behaviour compared with other health behaviours, such as smoking, for which the SoC theory has been applied (Adams & White 2005). Unlike other health-related behaviours such as smoking, changing exercise behaviours involves overall lifestyle and requires people ‘doing’ something rather than ‘not doing’ something. Committing to do something regularly may be a different sort of behavioural change, and may be structured in a complex way in relation to outcomes. Marttila *et al.* (1998) have classified physical activity in five categories: (a) occupational activity, (b) lifestyle activity, (c) recreation activity, (d) fitness activity and (e) sports activity. When respondents read the term exercise in each item, their interpretation may refer to several different types of physical activity. This may explain why some participants have high scores in different phases in the URICA-E2.

## Conclusion

The results of our study are potentially important in the global context of aging populations, increasingly sedentary lifestyles, and the financial burdens these will place on health resources. The results suggest that the utility of the URICA-E2 rather than the URICA-E (five-item stage specific categorizations) has the potential to identify individuals’ change profiles at different stages. Such profiles would be helpful both in predicting who would be more likely to change exercise behaviours, and also in matching behavioural change processes to meet individual needs. This means that the SoC construct can be further extended to consider alignment between the processes of change not only the stages of change themselves, but also with the change profiles that make up individuals’ stages. Further examination of the instrument in other populations may produce profiles which will aid in developing strategies to decrease the burden of healthcare costs related to sedentary lifestyles. In the validation process of the URICA-E2, we recommend using the categorical SoC measures to test criterion validity. We tested the instrument in a specific and relatively small sample. In future studies examining the psychometric properties of the URICA-E2, we recommend testing it in a more diverse population, particularly with regard to differences in age, gender and level of formal education.

Furthermore, further validation of the Action items and concurrent validity is required if this instrument is to be used in intervention studies. The main interest in using this continuous scale instead of the five-stage single-item scale is in its ability to configure profiles, not simply the stages of change, so that the theoretical ideas embedded in the Transtheoretical Model of Change can be tested in a rigorous fashion. Therefore, our findings, which indicate general agreement with the conceptual categories of the stages with the items in the scale, support the use of this continuous measure when a single-question instrument is not sufficient. However, one possible solution to remedy the redundancy of items in this 24-item scale is to re-configure the scale by selecting the two most clear statements for each stage and those with the highest factor loadings within each stage, and to test this shortened version for psychometric properties and validity to retain the continuous structure of the scale and for use in profile development.

## Notes

The *Journal of Advanced Nursing (JAN)* is an international, peer-reviewed, scientific journal. *JAN* contributes to the advancement of evidence-based nursing, midwifery and health care by disseminating high quality research and scholarship of contemporary relevance and with potential to advance knowledge for practice, education, management or policy. *JAN* publishes research reviews, original research reports and methodological and theoretical papers. For further information, please visit the journal web-site: http://www.journalofadvancednursing.com
